# An evaluation of inflammatory and endothelial dysfunction markers as determinants of peripheral arterial disease in those with diabetes mellitus

**DOI:** 10.1038/s41598-024-65188-w

**Published:** 2024-07-03

**Authors:** Sumera Zaib, Shabbir Ahmad, Imtiaz Khan, Yousef A. Bin Jardan, Gezahign Fentahun Wondmie

**Affiliations:** 1 Department of Basic and Applied Chemistry, Faculty of Science and Technology, University of Central Punjab, Lahore, 54590 Pakistan; 2 Department of Chemistry and Manchester Institute of Biotechnology, The University of Manchester, 131 Princess Street, Manchester, M1 7DN UK; 3 Department of Pharmaceutics, College of Pharmacy, King Saud University, P.O. Box 11451, Riyadh, Saudi Arabia; 4 Department of Biology, Bahir Dar University, P.O.Box 79, Bahir Dar, Ethiopia

**Keywords:** Interleukin-6, Interleukin-8, Peripheral arterial disease, Type 2 diabetes mellitus, Vascular cell adhesion molecule, Systems biology, Health care

## Abstract

The most serious long-term effects of diabetes is peripheral artery disease (PAD) which increases the chance of developing diabetic foot ulcers, gangrene and even lower limb amputation. The clinical manifestations of PAD which are typically not revealed until symptoms like intermittent claudication, rest pain and ischemic gangrene develop, are not present in majority of diabetes mellitus patients with PAD due to diabetic peripheral neuropathy. Therefore, current study is aimed to evaluate the inflammatory and endothelial dysfunction markers with their correlation to biomarkers that can help for in-time diagnosis and efficient prognosis of developing diabetes-associated PAD. Enzyme-linked immunosorbent assay was used to evaluate the interlukin-6, interlukin-8, intercellular adhesion molecule (ICAM) and vascular cell adhesion molecule (VCAM) in PAD with diabetes group, diabetic group and healthy individual group while biomarkers were measured by kit method. It was observed that serum IL-6, IL-8, ICAM and VCAM levels in type II diabetes mellitus (T2DM) with PAD patients were increased significantly (85.93, 597.08, 94.80 and 80.66) as compared to T2DM patients (59.52, 231.34, 56.88 and 50.19) and healthy individuals (4.81, 16.93, 5.55 and 5.16). The overall means for the parameters, IL-6, IL-8, ICAM, VCAM, urea, S/creatinine, CK-MB, AST, ALT, cholesterol, triglyceride, HDL, LDL, PT, aPTT, INR, HbA1C, and CRP within all groups were significantly (*P* < 0.05) different from each other. Therefore, it was concluded that the change in IL-6, IL-8, ICAM and VCAM can serve as an accurate diagnostic indicator and successful treatment.

## Introduction

Diabetes is a complicated chronic illness associated with persistent hyperglycemia due to impaired insulin production, impaired insulin sensitivity or both^1,2^. Increase in the incidence, disability and mortality rates of diabetes are being caused by the population’s changing dietary habits and lifestyle as well as the ongoing acceleration of the aging process, which has a significant impact on public health^3^. One of the most serious long-term effects of diabetes is peripheral artery disease (PAD) which increases the chance of developing diabetic foot ulcers, gangrene and even lower limb amputation^4^. Around 202 million individuals worldwide or 12–14% of the total population are reported to be affected with PAD^5,6^. It is more prevalent as people get older; affecting 10–25% of those over 55 and 40% of those over 80 and is linked to severe morbidity, mortality, and quality of life harm^7,8^. The clinical manifestations of PAD which are typically not revealed until symptoms like intermittent claudication, rest pain and ischemic gangrene appear, are not present in majority of diabetic patients with PAD because of diabetic peripheral neuropathy (DPN)^4,9^. The development of stenosis or aneurysms in non-coronary circulation can result from a number of pathophysiological events, although atherosclerosis is the most prevalent lesion that affects the aorta and its branches^5,10^.

The progressive buildup of lipids and inflammatory cells in the artery wall leads to the formation of atherosclerotic plaques^11,12^. The formation of atherosclerotic lesions is also greatly influenced by progressive endothelial dysfunction^13^. For the diagnosis of PAD, several plasma indicators of acute or chronic athero-inflammation, particularly inflammatory cytokines, acute indicators of endothelial dysfunction, subsets of mast cells, monocytes derived macrophages and all types of phase reactants play major role^14,15^. The classical inflammatory biomarkers have been found in numerous researches as vascular inflammation markers^16^. A non-specific marker of inflammation that rises with high concentrations of acute phase protein^17,18^, fibrinogen, a glycoprotein complex and a marker of thrombosis cascades, C-reactive protein (CRP) and inflammatory cytokines^19^. According to pathological stages and CV complications, it has been reported that circulating concentration of some cytokines such as CRP and interleukin (IL-6) are higher in PAD patients^20–23^. The levels of IL-6 and IL-8 are significantly higher in PAD patients^13,24–29^. IL-6 is linked to PAD severity^25,27,30^. In comparison to normal individual groups, PAD have been found to have higher levels of circulating soluble intercellular adhesion molecule (ICAM) and vascular cell adhesion molecule (VCAM)^13,26,28,29,31,32^. Particularly in the early phases of the disease or in diabetic patients, PAD frequently goes undiagnosed and untreated about 50% silent rates^10^. While some biomarkers have already found being used in clinical practice today, others need to be discovered and validated through research^13,33–35^.

The development and course of PAD are influenced by inflammation. Numerous potential inflammatory markers exists, such as established risk factors that promote atherogenicity via an inflammatory pathway. One of the biggest risk factors for PAD development is diabetes mellitus, which both directly and indirectly increases inflammatory pathways. Arterial hypertension caused by inflammatory markers, which affects around 80% of individuals with PAD may potentially be influenced by inflammation^36–39^. In fact vascular endothelial cells produce and express vascular cell adhesion molecule-1 in response to angiotensin II. Arterial smooth muscle cells (SMCs) release more pro-inflammatory cytokines, including interleukin (IL)-6, when exposed to angiotensin II Notably, every inflammatory molecule examined in these investigations contributes actively to peripheral pathogenesis rather than only acting as a signal of inflammation^40,41^. Nitric oxide (NO) released from endothelial cells is inhibited by CRP causing the severe atherosclerosis and loss of vascular flexibility. Increased levels of soluble intercellular adhesion molecule-1 (ICAM-1) have also been linked, independently, to the development of PAD, according to the Edinburgh Artery Study^42^.

This research is concentrated on chronic lower limb ischemia of atherosclerotic etiology known as lower extremity PAD. Our study is stressing the need for novel treatment targets, early diagnosis and prognostic markers and lower limb PAD due to the absence of accurate biomarkers for PAD assessment and crucial roles played by inflammation and vascular remodeling in the growth and rupture of atherosclerosis plaque.

## Material and methods

### Sample collection and preparation

10 mL of venous blood samples from DM, DM plus PAD, and healthy individuals were collected who visited the Mayo Teaching Hospital of King Edward Medical University (KEMU), Lahore, according to WHO guidelines following the consent of the patients involved in the research^43^. Blood samples were divided into two experimental groups and one control group. Diabetic blood samples (D-S1 and D-S2) were included in group 1 while blood samples from diabetics with PAD (DP-S1 and DP-S2) were included in group 2, and blood samples from healthy individuals were labeled as a control. Subsequently, subsamples were prepared by drawing 3 mL of blood from each tube into a gel vial with yellow cap for biochemical profiling, 2 mL in a citrated vial with blue cap for coagulation profiling, and 5 mL in a sterile vial with red cap in ethylenediaminetetraacetic acid (EDTA) for HbA1c analysis^44,45^.

### Interleukin-6 (IL-6)

Interleukin-6 is multifunctional cytokine that regulates immune responses, acute phase reactions and hematopoiesis and plays an important role in host defense mechanisms. Interleukin-6 was measured in serum samples via human IL-6 ELISA kit of Thermo Fischer Scientific. The serum was collected after centrifugation of blood at 4000 x*g* for 10 min. Lyophilized human IL-6 standard was reconstituted with sterile distilled water. Label seven sterile tubes, one for each standard marked as S1, S2, S3, S4, S5, S6 and S7. Prepared 1:2 serial dilutions for standard curve as follows; Take 225 μL of assay buffer (1x) into each tube. Pipette 225 μL of reconstituted standard (200 pg/ mL) into the first tube S1 and mix it gently. Take 225 μL of this dilution into 2nd tube S2 and mix thoroughly. Repeat serial dilutions 5 more times S33, S4, S5, S6 and S7. Lyophilized controls were reconstituted with distilled water. Pipette 100 μL of each standard dilution in the microwells.

Serum samples were diluted 1:2 (50 μL sample + 50 μL 1 × assay buffer) as add 50 μL assay buffer 1 × in samples well 50 μL serum, 50 μL high and low control and 100 μL in blank. An anti-human IL-6 antibody is adsorbed onto microwells of ELISA plate. Human IL-6 present in the serum or standard binds to antibodies adsorbed to the microwells. A 50 μL biotin-conjugated anti-human IL-6 antibody was added to all wells, incubated for 2 h at room temperature (18–25 °C) and bound to human IL-6 captured by the first antibody. Microwells were emptied and washed 4 times with wash buffer. A 100 μL of Streptavidin-HRP were added to all wells and incubated for 1 h at room temperature (18–25 °C). Microwells were emptied and washed 4 times with wash buffer. A 100 μL of TMB substrate solution was added to all wells and incubated at room temperature (18–25 °C) for 10 min. A 100 μL of stop solution was added to all wells and color density was measured at 450 nm in ELISA microplate reader^46^.

### Interleukin-8 (IL-8)

Interleukin-8/Neutrophil-Actvating Peptide-1 selectively stimulates the ability of neutrophils and T-lymphocytes to invade injured or inflamed tissue. Interleukin-8 was measured in serum samples via human IL-8 ELISA kit of Thermo Fischer Scientific. The serum was collected after centrifugation of blood at 4000 x*g* for 10 min. Lyophilized human IL-8 standard was reconstituted with sterile distilled water. Label seven sterile tubes, one for each standard marked as S1, S2, S3, S4, S5, S6 and S7. Prepare 1:2 serial dilutions for standard curve as follows; Take 225 μL of assay buffer (1x) into each tube. Pipette out 225 μL of reconstituted standard (200 pg/ mL) into the first tube S1 and mix it gently. Take 225 μL of this dilution into 2nd tube S2 and mix thoroughly. Repeat serial dilutions 5 more times S3, S4, S5, S6 and S7. Lyophilized controls were reconstituted with distilled water. Pipette 100 μL of each standard dilution in the microwells.

Serum samples were diluted 1:2 (50 μL sample + 50 μL 1 × assay buffer) as add 50 μL assay buffer 1 × in samples well, 50 μL serum, 50 μL high and low control and 100 μL in blank. An anti-human IL-8 antibody is adsorbed onto microwells of ELISA plate. Human IL-8 present in the serum or standard binds to antibodies adsorbed to the microwells. A 50 μL biotin-conjugated anti-human IL-8 antibody was added to all wells, incubated for 2 h at room temperature (18–25 °C) and bound to human IL-8 captured by the first antibody. Microwells were emptied and washed 3 times with wash buffer. A 100 μL of Streptavidin-HRP were added to all wells and incubated for 1 h at room temperature (18–25 °C). Microwells were emptied and washed 3 times with wash buffer. A 100 μL of TMB substrate solution was added to all wells and incubated at room temperature (18–25 °C) for 10 min. A 100 μL of stop solution was added to all wells and color density was measured at 450 nm in ELISA microplate reader^46^.

### Intercellular adhesion molecule-I (ICAM-I)

Intercellular Adhesion molecule-I (ICAM-I) is a member of the immunoglobulin supergene family and functions as a ligand for the lymphocyte function-associated antigen-1. ICAM-I was measured in serum samples via human ICAM-I ELISA kit of Thermo Fischer Scientific. The serum was collected after centrifugation of blood at 4000 x*g* for 10 min. Lyophilized human ICAM-I standard was reconstituted with sterile distilled water. Label four sterile tubes, one for each standard marked as S1, S2, S3 and S4. Prepare 1:2 serial dilutions for standard curve as follows; Take 225 μL of sample diluent into each tube. Pipette out 225 μL of reconstituted standard (1 = 100 ng/mL) into the first tube S1 and mix it gently. Take 225 μL of this dilution into 2nd tube S2 and mix thoroughly. Repeat serial dilutions 2 more times S3 and S4. Lyophilized controls were reconstituted with distilled water. Pipette 100  μL of each standard dilution in the microwells.

Serum samples were diluted 1:10 (10 μL sample + 90 μL sample diluent) as add 90 μL sample diluent in samples well with 10 μL serum, 10 μL high and low control and 100 μL in blank. An anti-human ICAM-I antibody is adsorbed onto microwells of ELISA plate. Human ICAM-I present in the serum or standard binds to antibodies adsorbed to the microwells. A 50 μL HRP-conjugated was added to all wells, incubated for 1 h at room temperature (18–25 °C) and bound to human ICAM-I captured by the first antibody. Microwells were emptied and washed 3 times with wash buffer. A 100 μL of Streptavidin-HRP were added to all wells and incubated for 1 h at room temperature (18–25 °C). Microwells were emptied and washed 3 times with wash buffer. A 100 μL of TMB substrate solution was added to all wells and incubated at room temperature (18–25 °C) for 10 min. A 100 μL of stop solution was added to all wells and color density was measured at 450 nm in ELISA microplate reader^47^.

### Vascular cell adhesion molecule-I (VCAM-I)

Vascular cell adhesion molecule-I (VCAM-I) or CD106 is a member of the immunoglobulin supergene family. VCAM-I was measured in serum samples via human VCAM-I ELISA kit of Thermo Fischer Scientific. The serum was collected after centrifugation of blood at 4000 x*g* for 10 min. Lyophilized human VCAM-I standard was reconstituted with sterile distilled water. Label six sterile tubes, one for each standard marked as S1, S2, S3, S4, S5 and S6. Prepare 1:2 serial dilutions for standard curve as follows; Take 225 μL of sample diluent into each tube. Pipette out 225 μL of reconstituted standard (1 = 100 ng/ mL) into the first tube S1 and mix it gently. Take 225 μL of this dilution into 2nd tube S2 and mix thoroughly. Repeat serial dilutions 4 more times S3, S4, S5 and S6. Lyophilized controls were reconstituted with distilled water. Pipette 100 μL of each standard dilution in the microwells.

Serum samples were diluted 1:50 (10 μL sample + 490 μL assay buffer) as add 490 μL assay buffer in 10 μL serum, then take 100 μL prediluted sample in all sample wells, 10 μL high and low control and 100 μL in blank. An anti-human VCAM-I antibody is adsorbed onto microwells of ELISA plate. Human VCAM-I present in the serum or standard binds to antibodies adsorbed to the microwells. A 50 μL conjugate-mixture was added to all wells, incubated for 2 h at room temperature (18–25 °C) and bound to human VCAM-I captured by the first antibody. Microwells were emptied and washed 3 times with wash buffer. A 100 μL of TMB substrate solution was added to all wells and incubated at room temperature (18–25 °C) for 10 min. A 100 μL of stop solution was added to all wells and color density was measured at 450 nm in ELISA microplate reader^47^.

### Estimation of glycan and glycosaminoglycan (HbA1c)

Measurement of hemoglobin-A1c was done on Beckman coulter system. Quantitative estimation of hemoglobin 1c concentration was performed in human whole blood. The HbA1c assay was carried out using the kit consisting of total hemoglobin reagent, HbA1c antibodies and HbA1c polyhepten and hemolyzing reagent^48^.

K2-EDTA whole blood was pre-treated with hemolyzing reagent in ratio of 1:100 and mixed well to obtain complete hemolysis of blood. Tetradecylammonium bromide (TTAB) in hemolyzing reagent eliminates the white blood cells. The concentration of both HbA1c and total hemoglobin were determined. HbA1c reagent was used to measure hemoglobin A1c concentration by turbidimetric immunoinhibition method. Hemoglobin A1c antibodies combine with HbA1c from sample to form soluble antigen–antibody complexes. Polyhepten then binds with excess antibodies and resulting agglutination complex was measured turbidimetrically at 450 nm^49^.

### Coagulation profiling

To evaluate coagulopathy, prothrombin time (PT) and activated partial thromboplastin time (aPTT) were measured according to the previous protocol^50^. For every 100 µL of citrated plasma sample, 200 µL of PT reagents were added and the time of coagulation was measured. For aPTT estimation, 100 µL of aPTT reagent was added to 100 µL of citrated plasma sample followed by incubation at 37 °C for 3 min. Subsequently, 100 µL of warmed calcium chloride was added to initiate the clotting cascade and time of clot formation was measured^50^.

### Measurement of C-reactive protein

The amperometric technique was used to measure the level of C-reactive protein in the 10 times diluted blood samples. After placing 30 µL of sample on immunosensor strips and 30 min of incubation, the electrodes were washed with PBS followed by dropping 50 µL of 0.5 mM ferrocyanide onto the electrodes. Ultimately, CRP concentration was determined using a standard calibration curve^51^.

### Serum creatinine

Serum samples were collected and creatinine levels were measured using Beckman Coulter AU analyzer. Jaffe’s method was employed which depicts the end results by the formation of red or orange yellow chromophores. All the reagents were already prepared. The kit method containing reagent 1 (R1) and reagent 2 (R2) was used consisting of sodium hydroxide and picric acid, respectively. The total volume of 1000 µL of reagents was added to 50 µL of serum sample in a 4:1 (R1:R2) ratio followed by 3 min of incubation. After incubation, the absorbance was measured at 520/800 nm^52,53^.

### Blood urea nitrogen

The measurement of blood urea nitrogen (BUN) was carried out in serum samples of patients by Beckman Coulter AU analyzer. At first urease enzyme was added to the serum sample in order to hydrolyze urea in to ammonia and carbon dioxide. Subsequently, l-glutamate dehydrogenase catalyzed the conversion of ammonia and *α*-oxoglutarate to glutamate. In the whole process, the hydrolysis of a urea molecule was associated with the oxidation of two molecules of NADH. At the end the absorbance was determined at 340 nm which increases with the falling levels of NADH in the reaction mixture^54^.

### Creatine kinase MB

The plasma levels of creatine kinase-MB (CK-MB) were estimated by the kit method. For obtaining plasma, the blood samples were centrifuged for 15 min at 500 x*g* at room temperature. After obtaining plasma, both reagents were added according to the manufacturer’s instructions and the absorbance was recorded using a microplate reader^54^.

### Aspartate aminotransferase (AST) and alanine aminotransferase (ALT)

The levels of AST are measured in serum samples that were stored at 15–20 ℃ and analyzed by using Beckman Coulter AU analyzer. 88 mmol/L of Tris buffer (pH 7.80), 900 U/L of lactate dehydrogenase (LDH), 260 nmol/L of l-aspartate, 600 U/L of malate dehydrogenase (MDH), 0.22 mmol/L of NADH and 12 mmol/L of *α*-oxoglutarate were used to measure AST. In this reaction, aspartate and *α*-oxoglutarate were catalyzed by AST to form oxaloacetate (OA) and l-glutamate. OA was reduced by MDH into l-malate in the presence of NADH which was converted in to NAD^+^. At the end of this reaction, the activity of AST was measured at 340 nm. The absorbance was found to decrease due to increase in the consumption of NADH indicating enhanced AST activity^52^.

ALT levels were also evaluated via Beckman Coulter AU analyzer with all ALT reagents prepared beforehand. At first amino group transfer reaction was catalyzed by ALT in which alanine and *α*-oxoglutarate were converted into glutamate and pyruvate. The reaction was further continued by the action of LDH on pyruvate in the presence of NADH to form lactate and NAD^+^. Afterwards, the absorbance was measured at 340 nm indicating the consumption of NADH and ALT activity^52^.

### Lipid profile estimation

#### Total cholesterol

To evaluate the total cholesterol levels, isopropanol: NP40 v/v (9:1) was used to dissolve the sample. Subsequently, 10 µL/well of 100 U/mL of catalase solution and 40 µL/well of sample solution in a black 96-well microplate were added. The microplate was incubated for 15 min at 37 °C to remove any peroxidase in the reagent or sample. The second incubation for 15 min at 37 °C was carried out after the addition of 150 µL/well of 0.67 U/mL cholesterol esterase plus reagent A, containing 0.4 mM ADHP, 1.3 U/mL HRP, 0.1% Triton X-100, 5 mM cholic acid, 0.1 M potassium phosphate buffer (pH 7.4), into each well of the microplate. After the second incubation, the absorbance was measured at the excitation and emission wavelengths of 530 and 580 nm, respectively^55^.

#### Triglycerides

The GPO/POD method was used to determine triglyceride (TG) levels in the venous blood samples. Microbial lipases were added to the blood sample to initiate the hydrolysis of TGs yielding glycerol and free fatty acids (FFAs). Subsequently, glycerol was phosphorylated by glycerol kinase and glycerol-3-phosphate oxidase into glycerol-3-phosphate (G3P) and oxidizes G3P generating dihydroxyacetone phosphate (DAP) and hydrogen peroxide (H_2_O_2_), respectively. The end product will be red quinoneimine produced by the reaction of H_2_O_2_ with 4-aminoantipyrine and 4-chlorophenol. The absorbance measured will be proportional to the concentration of TGs in the sample evaluated^56^.

#### Low- and high-density lipoprotein

High-density lipoproteins (HDL) and low-density lipoproteins (LDL) were measured simultaneously by the homogeneous method^29^. For HDL, chylomicrons, LDL and very low-density lipoproteins (VLDL) were blocked by the formation of antigen–antibody complexes in the sample and HDL was measured by the enzymatic cholesterol.

In the case of LDL, two types of reagents were used to quantify LDL in the sample solution. At first, reagent 1, which contains cholesterol oxidase, cholesterol esterase, ascorbic acid, 4-amino-antipyrine, MES buffer (pH 6.3), peroxidase, and detergent 1, was added to each well with the sample solution. Two types of reactions occur which involve the solubilization of non-LDL proteins and degradation of released cholesterol by detergent 1 and enzymes, respectively. Secondly, reagent 2, containing detergent 2, MES buffer (pH 6.3), and *N*,*N*-bis-*m*-tolidine-disodium, was added to solubilize LDL which was later quantified^57^.

### Statistical analysis

International Business Machine (IBM)—Statistical Package for Social Sciences (SPSS) version 23 was used for data analysis. Data was evaluated for overall means for various parameters using ANOVA. Significant difference of different parameters among various groups were evaluated using the repeated measures ANOVA (Dunnett’s T3 Post Hoc Test). The results of renal function tests, cardiac function test, ALT, lipid profile, coagulation profile, HbA1c and C-reactive proteins were correlated by healthy individuals, participants with diabetes mellitus (DM) and those with DM plus peripheral arterial disease (PAD) using the Pearson’s Correlation Coefficient (r value).

### Ethical approval

The Research and Ethics Committee (Path/No.195/2021), Faculty of Pathology, King Edward Medical University, Lahore, Pakistan, approved the current study.

## Results

### Evaluation of inflammatory and endothelial dysfunction markers and metabolic characteristics in patients with type 2 diabetes mellitus (T2DM) plus peripheral arterial disease (PAD), T2DM and healthy individuals

The ANOVA revealed that the overall means for the parameters, IL-6, IL-8, ICAM, VCAM, urea, S/creatinine, CK-MB, AST, ALT, cholesterol, triglyceride, HDL, LDL, PT, aPTT, INR, HbA1C, and CRP within all groups were significantly (*P* < 0.05) different from each other.

However, repeated measures ANOVA (Dunnett’s T3 Post Hoc Test) between various groups revealed that the IL-6, IL-8, ICAM and VCAM in healthy persons were significantly lower than the all-other groups. The data presented in Table 1 shows the mean plus standard error mean of different groups. The results revealed that serum IL-6 level in type II diabetes mellitus (T2DM) with peripheral arterial disease (PAD) patients was increased significantly (85.93) as compared to T2DM patients (59.52) and healthy individuals (4.81). Serum IL-8 level in type II diabetes mellitus (T2DM) with peripheral arterial disease (PAD) patients was increased significantly (597.08) as compared to T2DM patients (231.34) and healthy individuals (16.93). Similarly, the results revealed that serum ICAM level in type II diabetes mellitus (T2DM) with peripheral arterial disease (PAD) patients was increased significantly (94.80) as compared to T2DM patients (56.88) and healthy individuals (5.55). Serum VCAM level in type II diabetes mellitus (T2DM) with peripheral arterial disease (PAD) patients was increased significantly (80.66) as compared to T2DM patients (50.19) and healthy individuals (5.16).

**Table 1:** The Mean ± SEM of the different groups which were examined during the 2018–2022.

Parameters	Healthy control	Type-II diabetes mellitus	Type-II diabetes mellitus with peripheral arterial disease	Overall
IL-6	4.81 ± 0.61^a^	59.52 ± 2.82^b^	85.93 ± 2.14^c^	63.25 ± 3.25
IL-8	16.93 ± 2.53^a^	231.34 ± 21.27^b^	597.08 ± 39.01^c^	358.78 ± 30.35
ICAM	5.55 ± 0.32^a^	56.88 ± 2.012^b^	94.80 ± 1.07^c^	64.66 ± 3.46
VCAM	5.16 ± 0.62^a^	50.19 ± 3.54^b^	80.66 ± 1.94^c^	56.42 ± 3.25
Urea	20.21 ± 2.62^a^	25.67 ± 1.80^a^	50.64 ± 2.39^b^	35.31 ± 1.93
S/creatinine	0.60 ± 0.07^a^	0.72 ± 0.05^a^	1.52 ± 0.11^b^	1.04 ± 0.07
CK-MB	12.93 ± 1.42^a^	15.83 ± 0.86^a^	32.62 ± 2.82^b^	22.43 ± 1.56
AST	19.92 ± 2.45^a^	25.35 ± 1.54^a^	46.78 ± 3.90^b^	33.50 ± 2.17
ALT	18.85 ± 2.48^a^	20.02 ± 1.63^a^	49.89 ± 4.74^b^	32.39 ± 2.66
Cholesterol	144.78 ± 11.91^a^	201.75 ± 8.45^b^	239.00 ± 7.70^c^	208.35 ± 6.19
Triglyceride	113.29 ± 5.51^a^	211.75 ± 19.94^b^	417.43 ± 44.92^c^	282.56 ± 24.197
HDL	53.42 ± 1.91^a^	42.89 ± 1.50^b^	47.37 ± 1.79	46.45 ± 1.09
LDL	106.00 ± 4.44	115.43 ± 2.34	118.75 ± 3.12	115.32 ± 1.82
PT	12.85 ± 0.14^a^	13.45 ± 0.214^a^	15.10 ± 0.52^b^	14.05 ± 0.25
aPTT	32.92 ± 0.07^a^	33.86 ± 0.35^b^	36.24 ± 0.72^c^	34.72 ± 0.36
INR	0.98 ± 0.01	1.01 ± 0.03	1.56 ± 0.45	1.24 ± 0.19
HbA1C	4.79 ± 0.17^a^	8.94 ± 0.39^b^	9.05 ± 0.27^b^	8.32 ± 0.26
CRP	0.50 ± 0.05^a^	1.43 ± 0.22^b^	8.59 ± 2.90^c^	4.29 ± 1.27

Different superscript shows the statistical difference (*P* < 0.05) between the groups.

The results represented that serum urea level in type II diabetes mellitus (T2DM) with peripheral arterial disease (PAD) patients was increased significantly (50.64) as compared to T2DM patients (25.67) and healthy individuals (20.21). Likewise serum creatinine level was elevated significantly in T2DM with PAD patients (1.52) as compared to T2DM patients (0.72) and healthy individuals (0.60). Cardiac enzymes (CK-MB and AST) shows significantly increased level in T2DM with peripheral arterial disease (PAD) patients (32.62 and 46.78) as compared to T2DM patients (15.83 and 25.35) and healthy individuals (12.93 and 19.92). The results in Table 1 demonstrated that serum liver enzymes (ALT and AST respectively) were also raised significantly in T2DM with peripheral arterial disease (PAD) patients (49.89 and 46.78) as compared to T2DM patients (20.02 and 25.35) and healthy individuals (18.85 and 19.92). Lipid profile (cholesterol, triglycerides, HDL, and LDL) showed significantly elevated level in T2DM with PAD patients (239.00, 417.43, 47.37 and 118.75) as compared to T2DM patients (201.75, 211.75, 42.89 and 115.43) and healthy individuals (144.78, 113.29, 53.42 and 106.00). The coagulation profile (PT, aPTT and INR) in the Table 1 represents the significantly elevated level in T2DM with PAD patients (15.10, 36.24 and 1.56) as compared to T2DM patients (13.45, 33.86 and 1.01) and healthy individuals (12.85, 32.92 and 0.98). The HbA1c level was significantly declined in T2DM patients (8.94) as compared to T2DM with PAD patients (9.05) and lower in healthy individuals (4.79). Serum CRP level was significantly inclined in T2DM with PAD (8.59) as compared to T2DM patients (1.43) and healthy individuals (0.50).

### Pearson’s correlation of inflammatory and endothelial dysfunction markers and metabolic characteristics overall in those with type 2 diabetes mellitus (T2DM) plus peripheral arterial disease (PAD), T2DM and healthy individuals

The result of correlation coefficient of inflammatory and endothelial dysfunction markers of overall in (Table 2) IL-6 × IL-8 (r = 0.587; *P* < 0.01), IL-6 × ICAM (r = 0.819; *P* < 0.01), IL-6 × VCAM (r = 0.716; *P* < 0.01), IL-8 × ICAM (r = 0.750; *P* < 0.01), IL-8 × VCAM (r = 0.642; *P* < 0.01), and ICAM × VCAM (r = 0.799; *P* < 0.01) had a statistical significantly positive correlation.

**Table 2:** The Pearson’s correlation of Inflammatory and endothelial dysfunction markers of overall which were examined during the 2018–2022.

Pearson’s correlation coefficient among inflammatory and endothelial dysfunction markers of overall	
Parameters	r value
IL-6 × IL-8	0.587**
IL-6 × ICAM	0.819**
IL-6 × VCAM	0.716**
IL-8 × ICAM	0.750**
IL-8 × VCAM	0.642**
ICAM × VCAM	0.799**

**Correlation is significant at the 0.01 level (2-tailed), *Correlation is significant at the 0.05 level (2-tailed) and NS for non-significant.

The result of correlation coefficient of inflammatory marker (IL-6) with biochemical markers of overall in (Table 3) IL-6 × urea (r = 0.587; *P* < 0.01), IL-6 × S/creatinine (r = 0.474; *P* < 0.01), and IL-6 × CKMB (r = 0.445; *P* < 0.01), IL-6 × AST (r = 0.417; *P* < 0.01), IL-6 × ALT (r = 0.429; *P* < 0.01), IL-6 × cholesterol (r = 0.490; *P* < 0.01), IL-6 × triglyceride (r = 0.410; *P* < 0.01), IL-6 × LDL (r = 0.267; *P* < 0.05), IL-6 × PT (r = 0.303; *P* < 0.01), IL-6 × aPTT (r = 0.344; *P* < 0.01), IL-6 × HbA1C (r = 0.452; *P* < 0.01), IL-6 × CRP (r = 0.247; *P* < 0.05) had a statistical significantly positive correlation. While IL-6 × HDL and IL-6 × INR had no significant correlation.

**Table 3:** The Pearson’s correlation of inflammatory marker (IL-6) with biochemical markers of overall which were examined during the 2018–2022.

Pearson’s correlation coefficient among inflammatory marker (IL-6) and biochemical markers of overall	
Parameters	r value
IL-6 × Urea	0.587**
IL-6 × S/creatinine	0.474**
IL-6 × CK-MB	0.445**
IL-6 × AST	0.417**
IL-6 × ALT	0.429**
IL-6 × Cholesterol	0.490**
IL-6 × Triglyceride	0.410**
IL-6 × HDL	-0.168 ^NS^
IL-6 × LDL	0.267*
IL-6 × PT	0.303**
IL-6 × aPTT	0.344**
IL-6 × INR	0.153 ^NS^
IL-6 × HbA1C	0.452**
IL-6 × CRP	0.247*

**Correlation is significant at the 0.01 level (2-tailed), *Correlation is significant at the 0.05 level (2-tailed) and NS for non-significant.

The result of correlation coefficient of inflammatory marker (IL-6) with biochemical markers of overall in (Table 4) IL-8 × urea (r = 0.583; *P* < 0.01), IL-8 × S/creatinine (r = 0.521; *P* < 0.01), and IL-8 × CKMB (r = 0.458; *P* < 0.01), IL-8 × AST (r = 0.522; *P* < 0.01), IL-8 × ALT (r = 0.559; *P* < 0.01), IL-8 × cholesterol (r = 0.473; *P* < 0.01), IL-8 × triglyceride (r = 0.537; *P* < 0.01), IL-8 × PT (r = 0.425; *P* < 0.01), IL-8 × aPTT (r = 0.355; *P* < 0.01), IL-8 × HbA1C (r = 0.343; *P* < 0.01), IL-8 × CRP (r = 0.396; *P* < 0.05) had a statistical significantly positive correlation. While IL-8 × HDL, IL-8 × LDL and IL-8 × INR had no significant correlation.

**Table 4:** The Pearson’s correlation of inflammatory marker (IL-6) with biochemical markers of overall which were examined during the 2018–2022.

Pearson’s correlation coefficient among inflammatory marker (IL-8) and biochemical markers of overall	
Parameters	r value
IL-8 × Urea	0.583**
IL-8 × S/ Creatinine	0.521**
IL-8 × CK-MB	0.458**
IL-8 × AST	0.522**
IL-8 × ALT	0.559**
IL-8 × Cholesterol	0.473**
IL-8 × Triglyceride	0.537**
IL-8 × HDL	-0.065^NS^
IL-8 × LDL	0.126^NS^
IL-8 × PT	0.425**
IL-8 × aPTT	0.355**
IL-8 × INR	0.073^NS^
IL-8 × HbA1C	0.343**
IL-8 × CRP	0.396**

**Correlation is significant at the 0.01 level (2-tailed), *Correlation is significant at the 0.05 level (2-tailed) and NS for non-significant.

The result of correlation coefficient of endothelial dysfunction marker (ICAM) with biochemical markers of overall in (Table 5) ICAM × urea (r = 0.632; *P* < 0.01), ICAM × S/creatinine (r = 0.552; *P* < 0.01), and ICAM × CKMB (r = 0.485; *P* < 0.01), ICAM × AST (r = 0.498; *P* < 0.01), ICAM × ALT (r = 0.470; *P* < 0.01), ICAM × cholesterol (r = 0.523; *P* < 0.01), ICAM × triglyceride (r = 0.503; *P* < 0.01), ICAM × LDL (r = 0.224; *P* < 0.05), ICAM × PT (r = 0.307; *P* < 0.01), ICAM × aPTT (r = 0.346; *P* < 0.01), ICAM × HbA1C (r = 0.441; *P* < 0.01), ICAM × CRP (r = 0.275; *P* < 0.05) had a statistical significantly positive correlation. While ICAM × HDL and ICAM × INR had no significant correlation.

**Table 5:** The Pearson’s correlation of endothelial dysfunction marker (ICAM) with biochemical markers of overall which were examined during the 2018–2022.

Pearson’s correlation coefficient among endothelial dysfunction marker (ICAM) and biochemical markers of overall	
Parameters	r value
ICAM × Urea	0.632**
ICAM × S/ Creatinine	0.552**
ICAM × CK-MB	0.485**
ICAM × AST	0.498**
ICAM × ALT	0.470**
ICAM × Cholesterol	0.523**
ICAM × Triglyceride	0.503**
ICAM × HDL	-0.158 ^NS^
ICAM × LDL	0.224*
ICAM × PT	0.307**
ICAM × aPTT	0.346**
ICAM × INR	0.097 ^NS^
ICAM × HbA1C	0.441**
ICAM × CRP	0.275**

**Correlation is significant at the 0.01 level (2-tailed), *Correlation is significant at the 0.05 level (2-tailed) and NS for non-significant.

The result of correlation coefficient of endothelial dysfunction marker (VCAM) with biochemical markers of overall in (Table 6) VCAM × urea (r = 0.499; *P* < 0.01), VCAM × S/creatinine (r = 0.464; *P* < 0.01), and VCAM × CKMB (r = 0.486; *P* < 0.01), VCAM × AST (r = 0.358; *P* < 0.01), VCAM × ALT (r = 0.394; *P* < 0.01), VCAM × cholesterol (r = 0.452; *P* < 0.01), VCAM × triglyceride (r = 0.474; *P* < 0.01), VCAM × LDL (r = 0.340; *P* < 0.05), VCAM × PT (r = 0.243; *P* < 0.01), VCAM × aPTT (r = 0.247; *P* < 0.01), VCAM × HbA1C (r = 0.489; *P* < 0.01), VCAM × CRP (r = 0.244; *P* < 0.05) had a statistical significantly positive correlation. While VCAM × HDL and VCAM × INR had no significant correlation.

**Table 6:** The Pearson’s correlation of endothelial dysfunction marker (VCAM) with biochemical markers of overall which were examined during the 2018–2022.

Pearson’s correlation coefficient among endothelial dysfunction marker (VCAM) and biochemical markers of overall	
Parameters	r value
VCAM × Urea	0.499**
VCAM × S/ Creatinine	0.464**
VCAM × CK-MB	0.486**
VCAM × AST	0.358**
VCAM × ALT	0.394**
VCAM × Cholesterol	0.452**
VCAM × Triglyceride	0.474**
VCAM × HDL	-0.049^NS^
VCAM × LDL	0.340**
VCAM × PT	0.243*
VCAM × aPTT	0.247*
VCAM × INR	0.089 ^NS^
VCAM × HbA1C	0.489**
VCAM × CRP	0.244*

**Correlation is significant at the 0.01 level (2-tailed), *Correlation is significant at the 0.05 level (2-tailed) and NS for non-significant.

### Diagnostic performance of inflammatory and endothelial dysfunction markers in PAD + T2DM

**Figure 1:**
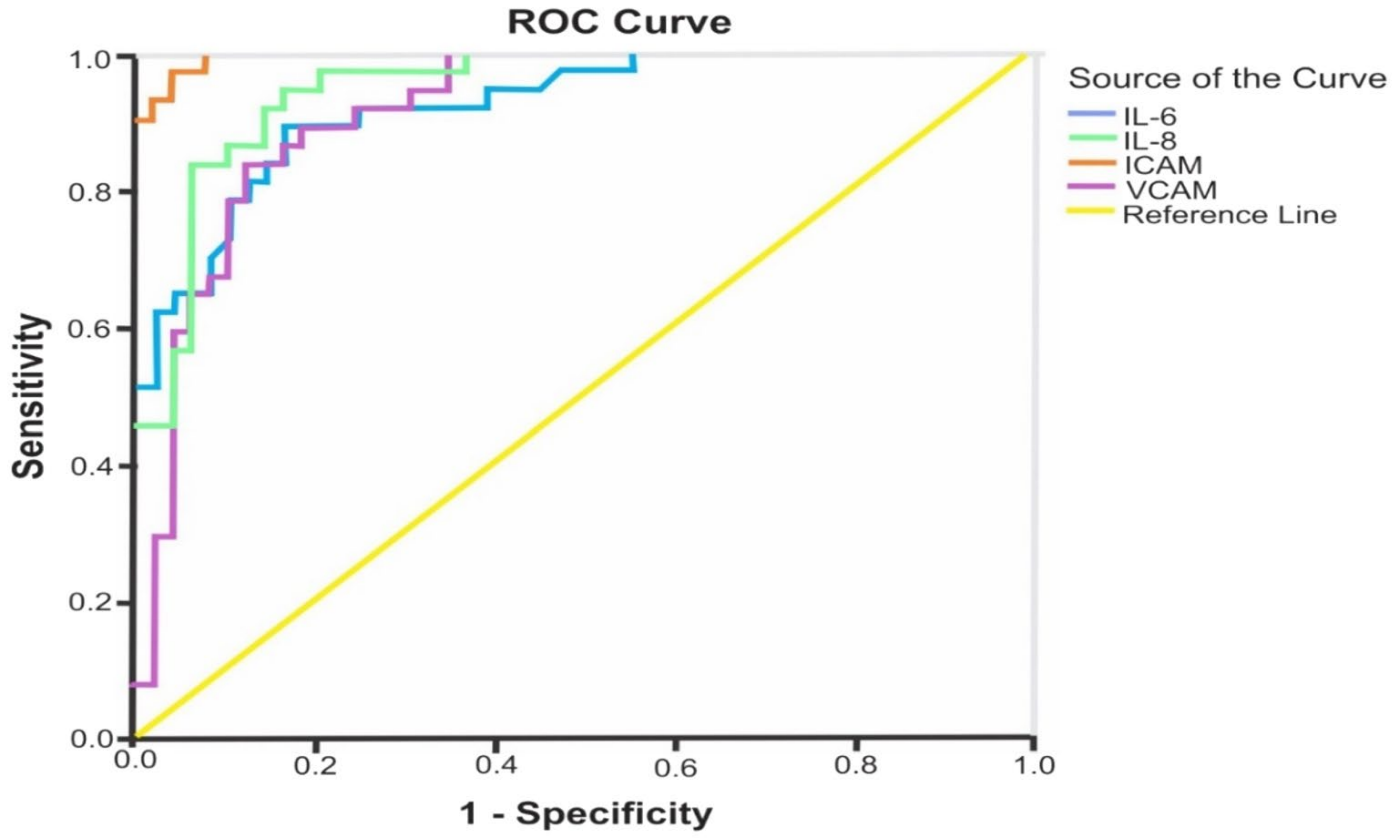
Receiver operating characteristics (ROC) for inflammatory and endothelial dysfunction markers.

The ROC curves represent a correct diagnostic performance for all four inflammatory and endothelial dysfunction markers. The area under curve (AUC, s) for IL-6, IL-8, ICAM and VCAM were higher than 0.8 (Fig. 1). AUCs for two inflammatory markers and two endothelial dysfunction markers were highly statistically significant (*p* < 0.005, 0.001) (Table 7).

**Table 7:** The diagnostic test performance for inflammatory and endothelial dysfunction markers in PAD + T2DM.

Test result variables (s)	AUC	Std. Error^a^	Asymptotic Sig.^b^	Asymptotic 95% confidence interval	
				Lower bound	Upper bound
IL-6	.924	.028	.000	.870	.978
IL-8	.949	.022	.000	.906	.991
ICAM	.999	.002	.000	.996	1.000
VCAM	.917	.030	.000	.858	.976

AUC, area under curve; CI, confidence interval.

AUC values for above all markers are significantly higher than 0.8 which is indicative of the accuracy of sensitivity analysis of a good model.

## Discussion

T2DM increases the susceptibility to complex pro-thrombotic state where endothelial dysfunction, platelet hyperactivity, changes to coagulation cascade and chronic low-grade inflammation play significant role^58^. Inflammatory biomarkers play important role in atherosclerosis^59^. IL-6 is released by fibroblasts, endothelial cells, macrophages, B-lymphocytes and Th2 lymphocytes and is pivotal in developing inflammatory response in PAD with diabetes^60^. IL-6 has played important role in the genesis of atherosclerosis and releasing of IL-8 by endothelial cells and macrophages^61^.

Numerous studies have examined the role of IL-6 in the pathophysiology of atherosclerosis particularly examining the relationship between the inflammatory process and peripheral artery disease (PAD). Numerous inflammatory molecules, sometimes referred to as acute-phase proteins, including CRP, fibrinogen synthesis, complement factor release, and serum amyloid A formation, are known to be stimulated by IL-6 in the liver^61,62^. IL-6 promotes the synthesis of MCP-1 and IL-8 by macrophages and endothelial cells, among other functions in the development and upkeep of atherosclerotic plaque. Additionally, IL-6 seems to be able to boost the synthesis of ICAM-1 by SMCs and increases the release of chemokines by the arterial wall's intimal cells. Furthermore, it facilitates leukocyte homing into atherosclerotic plaque. Lastly, IL-6 encourages SMCs to develop into foam cells. Research has demonstrated that IL-6 is a novel predictor of PAD and a valid marker for predicting the clinical course of the illness over a twelve-year span. Its function in PAD is actually well-defined, and evidence from community research indicates that it ought to be regarded as a predicting^63^.

Chronic consequences of diabetes mellitus (DM) include atherosclerosis in the arteries of the heart, brain and lower limbs. Leg ulcers and amputations can result from these issues that damage the lower limb arteries which is called PAD. Its burden is steadily increasing and contributes to diabetic’s mortality^64^. Peripheral artery disease (PAD) typically coexists with the conditions like diabetes, cardiac disease and respiratory illness. It is linked to the gradual loss of limb; claudication and gangrene witch ultimately lowers quality of life^65^. As a result, evaluation of a number of inflammatory biomarkers for the early diagnosis of PAD, in order to lower the risk of mortality and enhance their quality of life, is crucial.

Greater the exposure of the biomarkers IL-6 and IL-8 has resulted in a collection of research data on the role of interleukins (ILs) in PAD, specifically in relation to glycoproteins acting as proinflammatory substances that influence blood vessel walls when atherosclerotic plaque development is initiated and how it develops. The acute phase protein known as C-reactive protein (CRP) originates from liver cells. The secretion of interlukin-6 by macrophages and T-cells promotes its release^60^. The release of IL-8 from monocytes and macrophages depends critically on stimulated inflammatory circumstances. It is intriguing to observe that polymorph nuclear leukocytes significantly produce IL-8^66^. Significant associations between IL-18, P-selectin, VCAM and other inflammatory markers including IL-1, IL-6 and CRP as well as adhesion molecules like ICAM^67^. In our study, the results revealed that serum IL-6 level in type II diabetes mellitus (T2DM) with peripheral arterial disease (PAD) patients was increased significantly (85.93) as compared to T2DM patients (59.52) and healthy individuals (4.81). Serum IL-8 level in type II diabetes mellitus (T2DM) with peripheral arterial disease (PAD) patients was increased significantly (597.08) as compared to T2DM patients (231.34) and healthy individuals (16.93).

The susceptibility to exhibit continuous atherosclerosis progression is one of the primary characteristics of peripheral artery disease. It is generally known that inflammation plays a crucial role in the emergence of atherosclerosis. However, there is no previously reported inflammatory biomarker for the identification of the risk of stratification or conclusive diagnosis of PAD^68^. T2DM has significant higher levels of ICAM and VCAM compared to controls, an adhesion molecule considered to play a role in atherosclerosis progression^69^. Our results agree with earlier study, ICAM and VCAM were all found to be positively linked with DM in a prior study by Bluher^70^ as compared to hyperglycemic and impaired glucose tolerant patients. A more recent study reported a rise in VCAM blood concentration of PAD patients^71^. According to another recent report, VCAM is a marker that is pathogenically related to diabetic nephropathy and is predictive of it^68^. The results of our study revealed that serum ICAM level in type II diabetes mellitus (T2DM) with peripheral arterial disease (PAD) patients was increased significantly (94.80) as compared to T2DM patients (56.88) and healthy individuals (5.55). Serum VCAM level in type II diabetes mellitus (T2DM) with peripheral arterial disease (PAD) patients was increased significantly (80.66) as compared to T2DM patients (50.19) and healthy individuals (5.16).

To counteract the inflammation caused by other cytokines, IL-6 (as adipokine) levels gradually rise in DM, yet this can result in inflammation of vascular cells that leads to atherosclerosis^72^. Adipocytes in adipose tissue release IL-6 and IL-8 in obese individuals which induces insulin resistance particularly in hepatocytes and results in DM^73^. Due to the increased levels of cytokines and stress in DM, the levels of intercellular adhesion molecule (ICAM) and vascular cellular adhesion molecules (VCAM) are also increased. They are highly expressed in endothelial cells of arteries and trigger the release of new cytokines, which erodes the lining of the blood vessels^74^. IL-6, IL-8, ICAM and VCAM are rapidly rising in PAD causes the complications. So they induce endothelial abnormalities and harm the vascular system.

The levels of renal markers (BUN and serum creatinine) were also abnormally raised in DM plus PAD than in DM. BUN and creatinine are metabolic waste products that are normally excreted from kidneys, but under pathological conditions, they accumulate in the blood plasma^74^. In diabetics, the plasma level of creatinine drops due to decreased skeletal muscle volume; however, it rises in vascular disease indicating renal dysfunction and may increase or worsen atherosclerosis^75,76^. In critical limb ischemia, the level of BUN is also increased where it serves as a diabetic risk factor, a biomarker for hemodynamic changes, and an indicator of survival chances in patients^77^. In our study, the result of correlation coefficient of inflammatory markers (IL-6, IL-8) and endothelial dysfunction marker (ICAM, VCAM) with biochemical marker urea and creatinine of overall (IL-6, IL-8, ICAM, VCAM × urea, creatinine) had a statistical significantly positive correlation.

CRP is an indicator of cardiac dysfunction and functions as an acute phase reactant in blood plasma^78^. Its production is stimulated by an IL-6 mediated inflammatory signal in hepatocytes and adipose tissues; therefore, enhanced levels represent an inflammation or tissue injury^79,80^. As the level of CRP rises in blood so does the progression of atherosclerosis. It is because being acute phase reactant it activates platelets, complements system, initiates thrombus formation, and modifies innate immunity^81^. In our study, the result of correlation coefficient of inflammatory markers (IL-6, IL-8) and endothelial dysfunction marker (ICAM, VCAM) with biochemical marker AST, CKMB and CRP of overall (IL-6, IL-8, ICAM, VCAM × AST, CKMB and CRP) had a statistical significantly positive correlation.

Persistent glycemic control and prognosis of the T2DM therapy are critically associated with the levels of HbA1c, where, they represent the mean blood levels of glucose over a period of 3–6 months^82,83^. HbA1c levels are essentially elevated in T2DM as demonstrated by our results and agree with previous research^82^. Therefore, HbA1c can serve as an independent determinant for investigating diabetics that have the chance to develop PAD^84^. The association of HbA1c with PAD is due to increased glucose concentration over a long period of time thus activating platelets and protein kinase C pathway that induces pro-inflammatory condition leading to enhanced oxidation stress and endothelial dysfunction. Moreover, this consistent hyperglycemic condition also causes stiffness in the arteries^83^. In our study, the result of correlation coefficient of inflammatory markers (IL-6, IL-8) and endothelial dysfunction marker (ICAM, VCAM) with biochemical marker glycolytic hemoglobin** (**HbA1c) of overall (IL-6, IL-8, ICAM, VCAM × HbA1c) had a statistical significantly positive correlation.

Furthermore, the PT and aPTT (coagulation profile) level doesn’t increase preferably in diabetics plus PAD as compared to the control group and diabetics group. Unlike, enhanced fibrinogen levels are reported in diabetic patients as compared to non-diabetics^85^. Furthermore, coagulation profile indicates the risk of atherothrombotic events, the main cause of the development of cardiovascular complications^86^. In our study, the result of correlation coefficient of inflammatory markers (IL-6, IL-8) and endothelial dysfunction marker (ICAM, VCAM) with coagulation markers (PT, aPTT) of overall (IL-6, IL-8, ICAM, VCAM × PT, APTT) had a statistical significantly positive correlation.

Liver function tests were performed to estimate the levels of AST and ALT. Both the enzyme levels were elevated in diabetics and diabetics plus PAD. The level of AST and ALT significantly increases among diabetics and non-diabetics (control). These results agree to those reported previously^87^. Contrarily, when diabetics develop PAD symptoms, marked elevation in the levels of AST and ALT occurs. Diabetes with PAD can also be linked with abnormalities in the lipid profile of patients. The levels of triglycerides are elevated while low levels of HDL were observed in our investigations which are in accordance with the literature reports^88^. In our study, the result of the correlation coefficient of inflammatory markers (IL-6, IL-8) and endothelial dysfunction marker (ICAM, VCAM) with biochemical markers (AST, ALT, cholesterol, triglycerides, LDL) of overall (IL-6, IL-8, ICAM, VCAM × AST, ALT, cholesterol, triglycerides, LDL) had a statistical significantly positive correlation.

Although the level of all the tested parameters IL-6, IL-8, ICAM, and VCAM increased in DM plus PAD except and HDL levels, which are somehow, decreased from the control values. Any variation in markers of interest in healthy individuals, diabetics and diabetics plus PAD can be easily observed from Tables 1, 2, 3, 4, 5 and 6. A remarkable increase in IL-6, IL-8, ICAM and VCAM depicts that these analytes can be utilized as biomarkers in the diagnosis and prognosis of PAD associated with DM.

The clinical manifestations of PAD include claudication and pain at rest which progresses to ulceration and gangrene in later stages. Nonetheless, diabetic neuropathy may also be a secondary cause of leg pain and functional impairment^89^. One tool for categorizing the clinical phases of symptomatic PAD is the Fontaine scale. Fontaine stages IIa and IIb have mild and moderate to severe impairment claudication, respectively; Fontaine stage-III patients have symptoms at rest; and Fontaine stage-IV patients have considerable tissue loss (ulcers or gangrene). Patients at Fontaine stage-I have PAD but are asymptomatic^90^. Significant elevation of inflammatory and endothelial dysfunction markers include IL-6, IL-8, ICAM and VCAM can diagnose PAD patients even at Fontaine stage-I. Along these lines, machine learning techniques could be helpful in combing many biomarkers with clinical and functional factors to provide prediction algorithms that are more accurate. This will result in more precise PAD risk assessment, earlier PAD diagnosis and more individualized medication or surgery regimens^91^.

## Conclusion

In summary, diabetics can progressively develop PAD that damages their vascular system and increases the risk of cardiovascular disorders and lower limb amputations. Despite the incidence of PAD, poor therapeutic strategies have been used due to the lack of knowledge about the PAD clinical manifestations and limited markers for its early diagnosis, progression and prognostic evaluation in targeted manner. Keeping these problems in mind, we investigated a number of inflammatory and endothelial dysfunction markers along with biomarkers and found drastic alterations in the levels of inflammatory and endothelial dysfunction markers and some biomarkers in diabetics plus PAD as compared to diabetics only. These inflammatory and endothelial dysfunction markers include IL-6, IL-8, ICAM and VCAM. Among these biomarkers, marked increase in IL-6, IL-8, ICAM and VCAM were significantly higher in diabetics plus PAD as compared to DM and healthy individuals. In order to prevent the PAD in patients with DM, doctors may view these characteristics as useful diagnostic and prognostic criteria. The patients can be advised by clinicians to check their IL-6, IL-8, ICAM and VCAM from clinical pathology laboratory.

The current research also pointed out the interesting objectives particularly considering that vasoactive drugs commonly used in PAD treatment proved not to fulfill all the therapeutic targets. Further research is needed on additional inflammatory biomarkers in PAD to improve the database for their mechanisms and on novel effective therapies.

## Data Availability

All data generated or analyzed during this study are included in the published article.
